# Activation of the aryl hydrocarbon receptor by clozapine induces preadipocyte differentiation and contributes to endothelial dysfunction

**DOI:** 10.1177/02698811211055811

**Published:** 2022-01-03

**Authors:** K Fehsel, K Schwanke, BA Kappel, E Fahimi, E Meisenzahl-Lechner, C Esser, K Hemmrich, T Haarmann-Stemmann, G Kojda, C Lange-Asschenfeldt

**Affiliations:** 1 Neurobiochemical Research Unit, Department of Psychiatry, Medical Faculty, Heinrich Heine University Düsseldorf, Düsseldorf, Germany; 2 Leibniz Research Institute for Environmental Medicine (IUF), Düsseldorf, Germany; 3 Department of Internal Medicine I, University Hospital Aachen, RWTH Aachen University, Aachen, Germany; 4 Institute for Pharmacology and Clinical Pharmacology, Heinrich Heine University Düsseldorf, Düsseldorf, Germany; 5 Department of Plastic Surgery and Hand Surgery, Burn Center, University Hospital of the Aachen University of Technology, Aachen, Germany

**Keywords:** Clozapine, Cyp1A1, luciferase reporter assay, myography, vasodilatation, adipogenesis, aryl hydrocarbon receptor

## Abstract

**Background:**

The superior therapeutic benefit of clozapine is often associated with metabolic disruptions as obesity, insulin resistance, tachycardia, higher blood pressure, and even hypertension.

**Aims:**

These adverse vascular/ metabolic events under clozapine are similar to those caused by polycyclic aromatic hydrocarbons (PAHs), and clozapine shows structural similarity to well-known ligands of the aryl hydrocarbon receptor (AhR). Therefore, we speculated that the side effects caused by clozapine might rely on AhR signaling.

**Methods:**

We examined clozapine-induced AhR activation by luciferase reporter assays in hepatoma HepG2 cells and we proved upregulation of the prototypical AhR target gene Cyp1A1 by realtime-PCR (RT-PCR) analysis and enzyme activity. Next we studied the physiological role of AhR in clozapine’s effects on human preadipocyte differentiation and on vasodilatation by myography in wild-type and AhR-/- mice.

**Results:**

In contrast to other antipsychotic drugs (APDs), clozapine triggered AhR activation and Cyp1A1 expression in HepG2 cells and adipocytes. Clozapine induced adipogenesis via AhR signaling. After PGF2α-induced constriction of mouse aortic rings, clozapine strongly reduced the maximal vasorelaxation under acetylcholine in rings from wild-type mice, but only slightly in rings from AhR-/- mice. The reduction was also prevented by pretreatment with the AhR antagonist CH-223191.

**Conclusion:**

Identification of clozapine as a ligand for the AhR opens new perspectives to explain common clozapine therapy-associated adverse effects at the molecular level.

## Introduction

The antipsychotic drug (APD) clozapine is effective even in treatment-refractory schizophrenia, but it also shows common adverse effects which occur to a lesser extend with other APDs. This difference to other APDs may be associated with additional biochemical mechanisms apart from modulation of the activity of various neuronal receptors as such receptors are targeted by other APDs as well. Accordingly, clozapine is taken up into the cell by a carrier-mediated process, although the direct transporter is still unknown (Dickens et al., 2018). Within the cell, unique components of clozapine’s chemical structure, that is a halogenated diazepine, might trigger specific cellular responses such as activation of the aryl hydrocarbon receptor (AhR). Our assumption that this ligand-activated transcription factor plays a key role in clozapine-triggered adverse effects is supported by the fact that AhR signaling causes weight gain, hyperglycemia, and hypertension (Chang et al., 2017; Rojas et al., 2021) too. Besides activation of detoxification processes, the AhR is also involved in cell differentiation, angiogenesis, and stress responses. Figure S1 gives an overview of the involvement of the AhR in several diseases. Moreover, proteomic pathway analysis revealed an altered AhR pathway in the hippocampus of people diagnosed with schizophrenia (Schubert et al., 2015) suggesting that an interaction of clozapine might contribute to its antipsychotic activity.

The AhR is a ligand-inducible, ubiquitous transcription factor that mediates cellular responses to dioxins, polycyclic aromatic hydrocarbons (PAHs), and related environmental chemicals. Upon ligand binding, the AhR dissociates from HSP90 and c-src-kinase, translocates to the nucleus, dimerizes with the AhR nuclear translocator (ARNT), and binds to dioxin-responsive elements (DRE) in the promoters of target genes to enforce transcription. Beside transcriptional upregulation of drug-metabolizing enzymes, the receptor activates multiple genomic as well as non-genomic pathways involved in stem cell differentiation, cell plasticity, and immunosuppression (Abel and Haarmann-Stemmann, 2010; Matsumura, 2009). In addition, PAH exposure as well as clozapine treatment increase the risk of bone marrow toxicity (Lee et al., 2004; Luster et al., 1985) and liver steatosis (Rojas et al., 2021; Von Wilmsdorff et al., 2014) as well as the incidence of diabetes, abdominal obesity (Lee et al., 2014; Teff and Kim, 2011), and hypertension (Alberich et al., 2019; Lee et al., 2012).

The aim of this study was to investigate whether clozapine might interact with the AhR in the cytoplasm resulting in activation of this receptor. Therefore, aripiprazole, haloperidol, olanzapine, and clozapine were screened for their potential AhR binding activity by using a DRE-driven luciferase reporter assay. As for effects on cellular functions, adipogenesis which is partially stimulated by AhR was assessed. Finally, effects on vascular functions were investigated by monitoring acetylcholine-induced endothelium-dependent vasorelaxation.

## Materials and methods

### Animals

C57BL/6 mice (male, 3–4 months old, 24–28 g) were purchased from Janvier Labs (Le Genest-Saint-Isle, France). Transgenic AhR knockout mice (AhR^-/-^) in which exon 2 of the AhR is deleted (Schmidt et al., 1996) were bred and housed under specific pathogen free conditions in the animal facility of the IUF, Düsseldorf, Germany. Animals (*n* = 3–5 per cage) were housed in standard cages at a 12-h light/dark circle and received a standard mouse chow and acidified water (pH = 3–4) ad libitum. To avoid a possible influence of the oestrous cycle, we investigated male mice only. All animals were treated according to the guidelines for the use of experimental animals, as given by the German “Tierschutzgesetz” and the Directive 2010/63/EU of the European Parliament. The vasorelaxation studies were approved by the regional government (LANUV approval references: O45/87) and the Directive 2010/63/EU of the European Parliament.

### Drug preparations

Clozapine (generous gift from Novartis Pharmaceuticals, Nuremberg, Germany) and olanzapine were dissolved in dimethyl sulfoxide (DMSO) at a concentration of 200 mM, and then diluted further with ethanol to create a stock solution of 20 mM. Haloperidol and aripiprazole were solved in ethanol at a concentration of 100 mM. Concentrations were selected based on the median serum concentration of each APD (Jönsson et al., 2019). Serum concentration of clozapine (1µM) is at least 10-fold higher than that of olanzapine (80 nM), risperidone (70 µM), and haloperidol (7 nM). Therefore, concentrations for all APDs were in keeping with the concentrations used for treatment in patients. The AhR inhibitors α-naphthoflavone (α-NF, Sigma-Aldrich, Dreieich, Germany) and 3’methoxy-4’nitroflavone (MNF, generous gift from Dr. Imke Meyer, Symrise AG, Holzminden, Germany) as well as the green tea ingredient epigallocatechin-3-gallate (EGCG) were dissolved in DMSO. The AHR antagonist 1-Methyl-N-[2-methyl-4-[2-(2-methylphenyl)diazenyl]]phenyl-1H-pyrazole-5-carboxamide (CH223191) (Santa Cruz, Heidelberg, Germany) was dissolved in ethanol to a concentration of 10 mM. The final DMSO concentration was less than 1% (v/v) in all settings. All solutions were prepared immediately prior to use.

Clozapine is an atypical APD which interferes with the activity of dopamine, serotonin, and noradrenaline and acts as an antagonist at dopamine (D2), serotonin (5-HT2), and noradrenaline (alpha-2) receptors. Aripiprazole is an atypical APD which interferes with the activity of dopamine and serotonin and acts as an antagonist at dopamine (D2) and serotonin (5-HT1A) receptors. Olanzapine is an atypical APD which interferes with the activity of dopamine and serotonin and acts as an antagonist at dopamine (D2) and serotonin (5-HT2) receptors. Haloperidol is a typical APD which interferes with the activity of dopamine and acts as an antagonist at dopamine (D2) receptors.

### Luciferase reporter assays

HepG2 XRE reporter cells were provided by Lorenz Poellinger and Katharina Gradin (Karolinska Institute, Stockholm, Sweden); 2 × 10^5^ cells per well were seeded in six-well plates and cultured to ∼80% confluence (Rolsted et al., 2008). Cells were treated with clozapine (20 µM, 30 µM, or 40 µM), olanzapine or aripiprazole (0.5µM or 5µM) or haloperidol (5µM or 20µM) for 4 h and then lysed in 200 μl of lysis buffer (25 mM Tris-phosphate, pH 7.8, 2 mM dithiothreitol, 2 mM (1,2-cyclohexylenedinitrilo) tetraacetic acid, 10% (v/v) glycerol, and 1% (v/v) Triton X-100). Lysate (20 μl) was combined with 80 μl of Luciferase Reporter Substrate (Promega, Madison, WI) and luciferase activity measured with a TD-20e luminometer (Turner Systems, Sunnyvale, CA). Luciferase activity was normalized with respect to protein concentration, which was determined by the bicinchoninic acid (BCA) assay (ThermoFisher, Langenselbold, Germany).

### Ethoxyresorufin-O-deethylase (EROD) activity

For measuring CYP1A1 activities, 7-ethoxyresorufin (Sigma-Aldrich; solved in DMSO) was employed according to a protocol described by Rolsted et al. (2008). Shortly, HepG2 cells were plated into 12-well plates at a density of 1×10^6^ cells/ml, stimulated with clozapine, benzo(a)pyrene (BaP), MNF either alone or in combination; and incubated for 24 h. Then, serum-free media containing 2.5 µM 7-ethoxyresorufin and 10 µM dicumarol were applied to phosphate buffered saline (PBS)-washed HepG2 cells and resorufin formation kinetics were measured 20 min at 37°C at excitation and emission wavelengths of 544 nm and 590 nm on a Fluoroskan Ascent reader (Labsystems, Bornheim, Germany). All experiments were carried out three times in triplicate.

### Adipogenesis

All experiments had been approved by the Ethical Committee of the Aachen University of Technology. Human preadipocytes were isolated (Hemmrich et al., 2005) and seeded with a density of 3 × 10^4^ cells/cm². They were cultured until confluence in Dulbecco`s Modified Eagles`s Medium (DMEM)/F12 medium supplemented with 10% fetal calf serum (FCS) and basic fibroblast growth factor (bFGF) (10 ng/ml). Adipogenic conversion was then promoted for 14 days by changing medium to DMEM/F12 without serum addition, but supplemented with 66 nM insulin, 100 nM dexamethasone, 0.5 mM 3-isobutyl-1-methylxanthine (IBMX), 0.1 µg/ml pioglitazone, 1 nM triiodo-L-thyronine, and 10 µg/ml human transferrin. After 5 days of incubation, medium was used as before but without IBMX and pioglitazone for 9 more days. Clozapine (5 µM and 20 µM), the AhR antagonist αNF (10 µM), and the green tea extract epigallocatechin-3-gallate (EGCG) (10 µM) were added on days 1, 3, and 5 of differentiation. After 14 days of differentiation, adipogenic conversion was determined by measuring the activity of the fat cell specific enzyme glycerol-3-phosphate dehydrogenase (GPDH) (Hemmrich et al., 2005) and morphologically by Oil Red O staining.

### Vasorelaxation studies

Male C57BL6 mice as well as AhR^-/-^ mice were sacrificed by inhalation of either carbon dioxide or isoflurane. Aortic vascular reactivity was determined using water jacketed organ bath filled with 10 ml of Krebs-Henseleit buffer and gassed with 95% oxygen and 5% carbon dioxide through sintered-glass filters at a constant temperature of 37°C (Suvorava et al., 2015). Aortic constrictions were measured by calibrated Statham transducers (Ametek, Rochester, USA) and recorded (SE-120; ABB, Mannheim, Germany). Aortic rings were cut into 5 mm rings and mounted between two stainless steel triangles. Vascular tone was stabilized during equilibration under a resting tension of 9.81 mN. The maximal contractile response was evaluated by 80 mM KCl and the maximal contractile response to prostaglandin F_2α_ (PGF_2α_) was evaluated by addition of 10 µM. Intact endothelium was proven by maximal vasodilatation in response to 10 µM acetylcholine (ACh) after half-maximal preconstriction with 1.2 µM PGF_2α_. Assessment of endothelium-dependent vasodilation was then performed by adding accumulating concentrations of ACh (0.01–10 µM) following a second preconstriction with 1.2 µM PGF_2α_, either under vehicle conditions (0.005% DMSO) or following 20 min preincubation with the APDs haloperidol (1 µM) (*n* = 6), aripiprazole (1 µM) (*n* = 10), olanzapine (0.5 µM) (*n* = 6), or clozapine (1 µM) (n = 8). In another set of experiments, 10 µM of the AhR antagonist CH223191 was applied 40 min prior to addition of clozapine and endothelium-dependent vasodilation (*n* = 6). In addition, endothelium-independent vasorelaxation to nitric oxide (NO) was tested by adding the NO-donor diethylammonium (Z)-1-(N,N-diethylamino)diazen-1-ium-1,2-diolate (DEA/NONOate from Biomol (Hamburg, Germany) in a concentration-dependent manner (0.01–10 µM) under control conditions and after 20 min preincubation with clozapine (1 µM) (*n* = 8).

### PCR analysis

RNA was isolated from HepG2 cells and adipocytes using the RNeasy^®^ Protect Cell Mini Kit (Qiagen, Hilden, Germany). Gene expression was studied by quantitative real-time PCR and semiquantitative PCR as previously described (Haarmann-Stemmann et al., 2010; Fehsel et al., 2005). Quantitative RT-PCR measurements in HepG2 cells were standardized using actin as house-keeping control gene. For semiquantitative PCR analysis of adipogenic marker genes glyceraldehyde-3-phosphate-dehydrogenase (GAPDH) and actin were chosen as controls. In Tab. S1 and S2 the sequences of the primers are listed.

### Statistics

Data were expressed as mean ± SEM and analyzed using GraphPad Prism^®^ Version 6.07 (GraphPad Software Inc., San Diego, California, USA). Data were analyzed by either one-way analysis of variance (ANOVA) following Tukey’s multiple comparison test or by two-way ANOVA following Sidak multiple comparison tests. Values of *p* < 0.05 were considered significant.

## Results

### AhR activation

**Figure 1.**
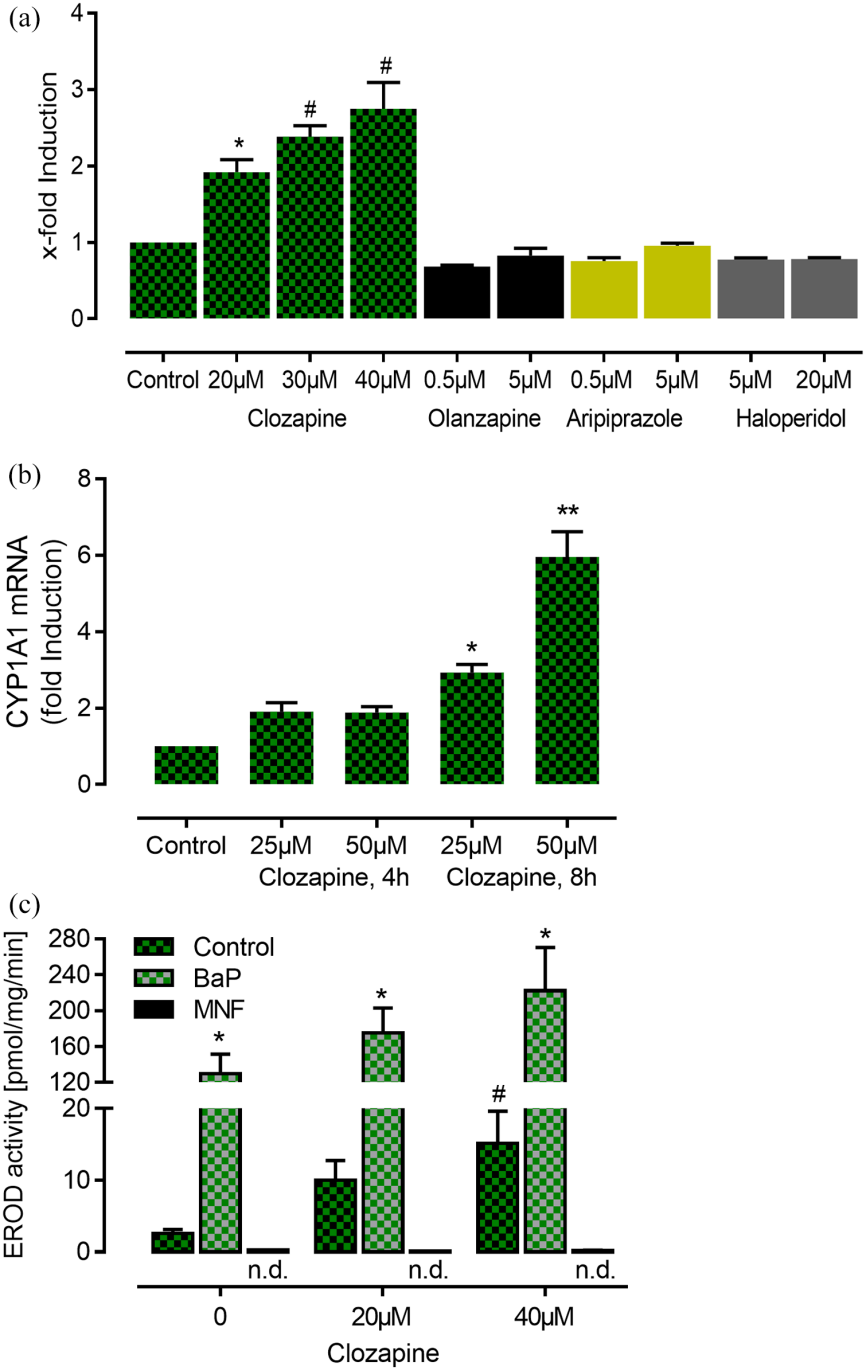
Clozapine activates the AhR and increases the expression and activity of CYP1A1 in HepG2 cells (bars represent SEM). (a) Clozapine concentration-dependently increases luciferase activity in cells stably transfected with dioxin-responsive elements, while other antipsychotic drugs have no effect (*p* < 0.0001, one-way ANOVA, *p = 0.0102, ^#^*p* < 0.0001 vs control, Tukey’s multiple comparisons test, *n* = 6, triplicates each). Of note, in comparison to Benzo(a)pyrene (BaP, 1 μM) which elicited a 18.7 ± 2.3-fold induction, the effect of clozapine appears to be rather moderate. (b) Clozapine concentration-dependently induces CYP1A1 mRNA expression (*n* = 7, *p* < 0.001, one-way ANOVA, *p = 0.0011 and **p < 0.0001 vs control, Tukey’s multiple comparisons test, *n* = 5, triplicates each). (c) Clozapine concentration-dependently induces CYP1A1 activity as measured by the ethoxyresorufin-O-deethylase (EROD) assay. Noteworthy, EROD activity induced by both concentrations of clozapine was completely abolished in the presence of MNF demonstrating that the AhR antagonist MNF completely reversed AhR agonist activity of clozapine. The combination of clozapine with Benzo(a)pyrene (BaP, 1 μM) resulted in a strong augmentation of EROD activity. In addition, MNF also abolished the small increase of EROD activity under control conditions in absence of clozapine (*p* < 0.0001, one-way ANOVA, *p < 0.005 BaP (1 μM) vs DMSO/ethanol control, ^#^*p* = 0.0421, 0 µM clozapine vs 40 µM clozapine, Tukey’s multiple comparisons test, *n* = 3, triplicates each, not indicated is the significant difference between clozapine alone and the combination of clozapine and BaP, *p* < 0.0002).

To investigate clozapine-induced AhR activation, we performed DRE-driven reporter assays using HepG2 reporter cells. Short treatment (4 h) of these cells with clozapine (20 µM–40 µM) resulted in a significant and concentration-dependent two- to threefold induction of luciferase activity indicating that clozapine directly activates AhR signaling (Figure 1(a)). However, the magnitude of this effect of clozapine is smaller than that of BaP (1 nM) which elicited a 18.7 ± 2.3-fold induction of luciferase activity. Additional luciferase reporter assays revealed that haloperidol, aripiprazole, and olanzapine did not activate the AhR (Figure 1(a)). While olanzapine tended to increase luciferase activity at a high concentration of 50 µM to 1.31 ± 0.076-fold induction, *p* > 0.05), the same concentration of haloperidol and aripiprazole did not (data not shown). Real-time PCR analyses revealed a significant time- and concentration-dependent increase of the mRNA expression of the prototype AhR target gene CYP1A1 (Figure 1(b)). Accordingly, catalytic activity of CYP1A1, determined via O-deethylation of 7-ethoxyresorufin also increased in a dose-dependent manner (Figure 1(c)). Interestingly, clozapine plus BaP co-exposure produced additive/synergistic effects on EROD activity, indicating that clozapine did not antagonize the BaP response, as often observed for low-affinity ligands (Abel and Haarmann-Stemmann, 2010). Noteworthy, EROD activity induced by both concentrations of clozapine was completely abolished in the presence of MNF demonstrating that the AhR antagonist MNF completely reversed AhR agonist activity of clozapine. In addition, MNF also abolished the small increase of EROD activity under control conditions in absence of clozapine. Further analysis of gene expression in clozapine-treated HepG2 cells revealed only slight induction of other genes upregulated by AhR activation like cytochrome P450 monooxygenase 1A2 (Cyp1A2), plasminogen activator inhibitor (PAI2), nuclear factor-erythroid 2 p45-related factor 2 (NRF2), NAD(P)H:quinone oxidoreductase 1 (NQO1) after 4 and 8 h. After 16 h of treatment, the expression of ER stress transcription factor activating transcription factor 4 (ATF4) and vascular endothelial growth factor (VEGF) were significantly induced (Tab. S3). Both genes were previously shown to be triggered by the AhR in HepG2 cells under glucose deprivation (Terashima et al., 2013).

### Adipogenesis

**Figure 2.**
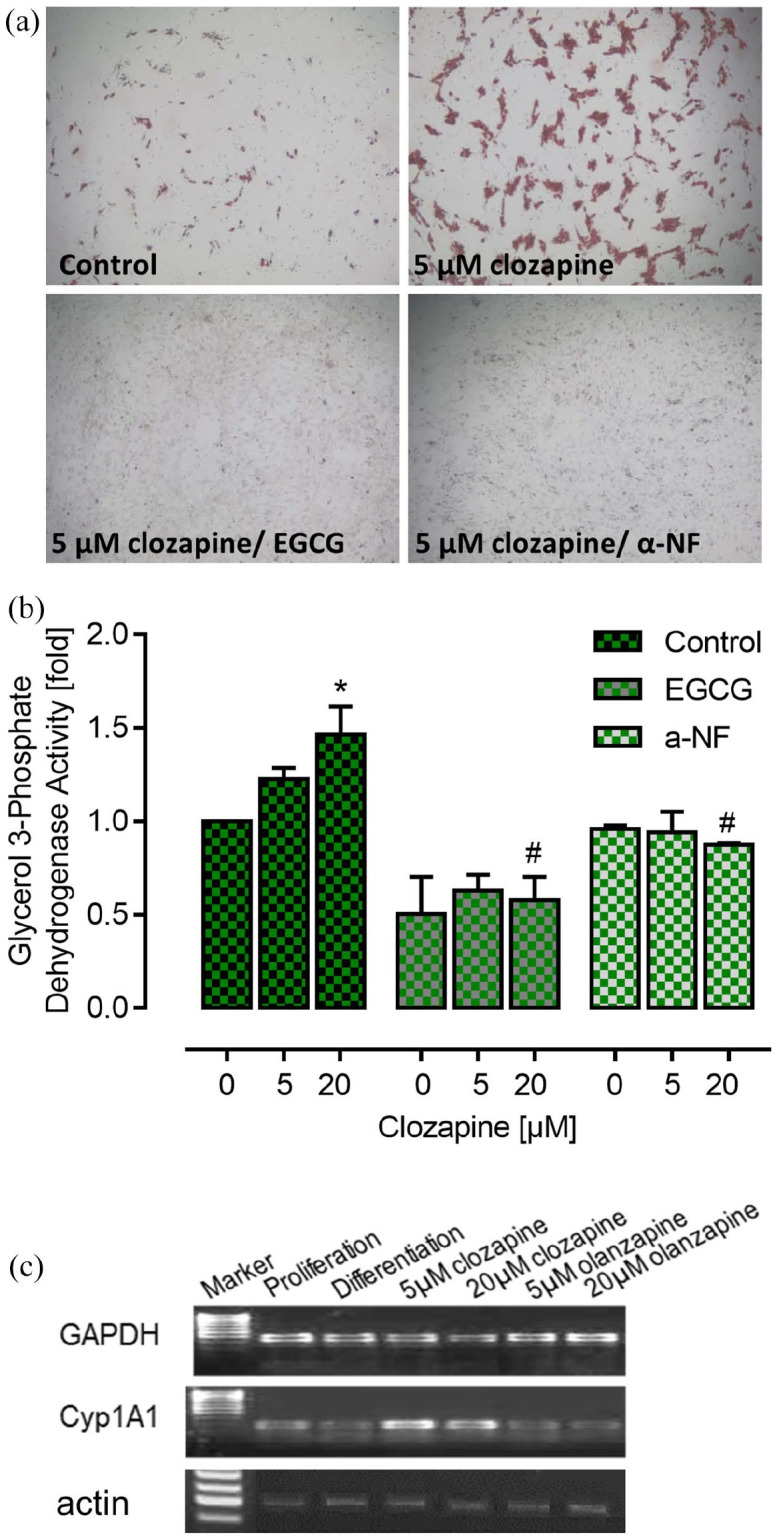
Effect of clozapine on the activity of glycerol 3-phosphate dehydrogenase as a marker for the differentiation of preadipocytes. All data are related to the value obtained following incubation with dimethyl-sulfoxide/ethanol as a vehicle (0 µM clozapine) in the control group. (a) Representative pictures of cultured adipocytes stained with oil red which indicates differentiation to adipocytes following control conditions or incubation with clozapine, either alone or in combination with the aryl hydrocarbon receptor inhibitors epigallocatechin gallate (EGCG) or α-naphtoflavone (α-NF). (b) While clozapine increased the activity of glycerol 3-phosphate dehydrogenase in the control group (*p* < 0.0001, one-way ANOVA, *p < 0.05 vs vehicle, Tukey’s multiple comparison test, *n* = 5), this effect was completely inhibited after preincubation with EGCG or α-NF. In addition, both inhibitors significantly reduced the effect of 20 µM clozapine (*n* = 3–5, *p* < 0.0001, one-way ANOVA, ^#^*p* < 0.05 vs control value, Tukey’s multiple comparison test). (c) Cyp1A1 expression in differentiated PAs was proved by real-time PCR and semiquantitative PCR. Figure 2(c) shows representative gels with Cyp1A1 PCR products only after clozapine incubation (5 and 20 µM).

To test our hypothesis in a human physiological model system, we inhibited preadipocyte differentiation in clozapine-treated cells with the AhR antagonist α-NF and with the green tea extract EGCG which inhibits DNA binding of the AhR/ARNT complex (Palermo et al., 2005). Preadipocyte differentiation was significantly higher under clozapine treatment compared with standard differentiation, while combination with EGCG or α-NF showed the very effective inhibition of triglyceride (TG) accumulation (approximately 85%) (Figure 2(a)). Even standard differentiation was reduced with both agents, suggesting that one or more ingredients of the differentiation medium—probably triiodo-L-thyronine (T3) (Colombo et al., 2003)—activate the AhR as well. All GPDH findings depicted in Figure 2(b) were supported by identical morphological results, that is, fewer lipid vesicles and elongated cell shape under EGCG or α-NF treatment (Figure 2(a)). RT-PCR analysis of the treated preadipocytes revealed expression of the AhR target gene Cyp1A1 under clozapine but not under olanzapine treatment. Figure 2(c) shows the PCR products of one representative experiment including the house-keeping genes GAPDH and ß-actin. In Figure S4, further PCR analyses revealed no expression changes in most of the lipogenic genes under clozapine and/or EGCG. This surprising result is in line with the findings that their maximum expression is on day 6 of differentiation. Then mRNA levels decrease (Salehpour et al., 2020). The expression of hormone sensitive lipase (HSL)—a marker enzyme of white adipocytes—was inhibited by EGCG after 14 days of differentiation, while clozapine even increased its expression during adipogenesis.

In order to find another potential trigger of adipogenesis, we stained the differentiated preadipocytes for superoxide production. Only clozapine-treated preadipocytes showed strong staining (data not shown), indicating that AhR-induced mitochondrial superoxide production (Sun et al., 2021) might contribute to preadipocyte differentiation (Kim et al., 2014).

### Vasorelaxation

**Figure 3.**
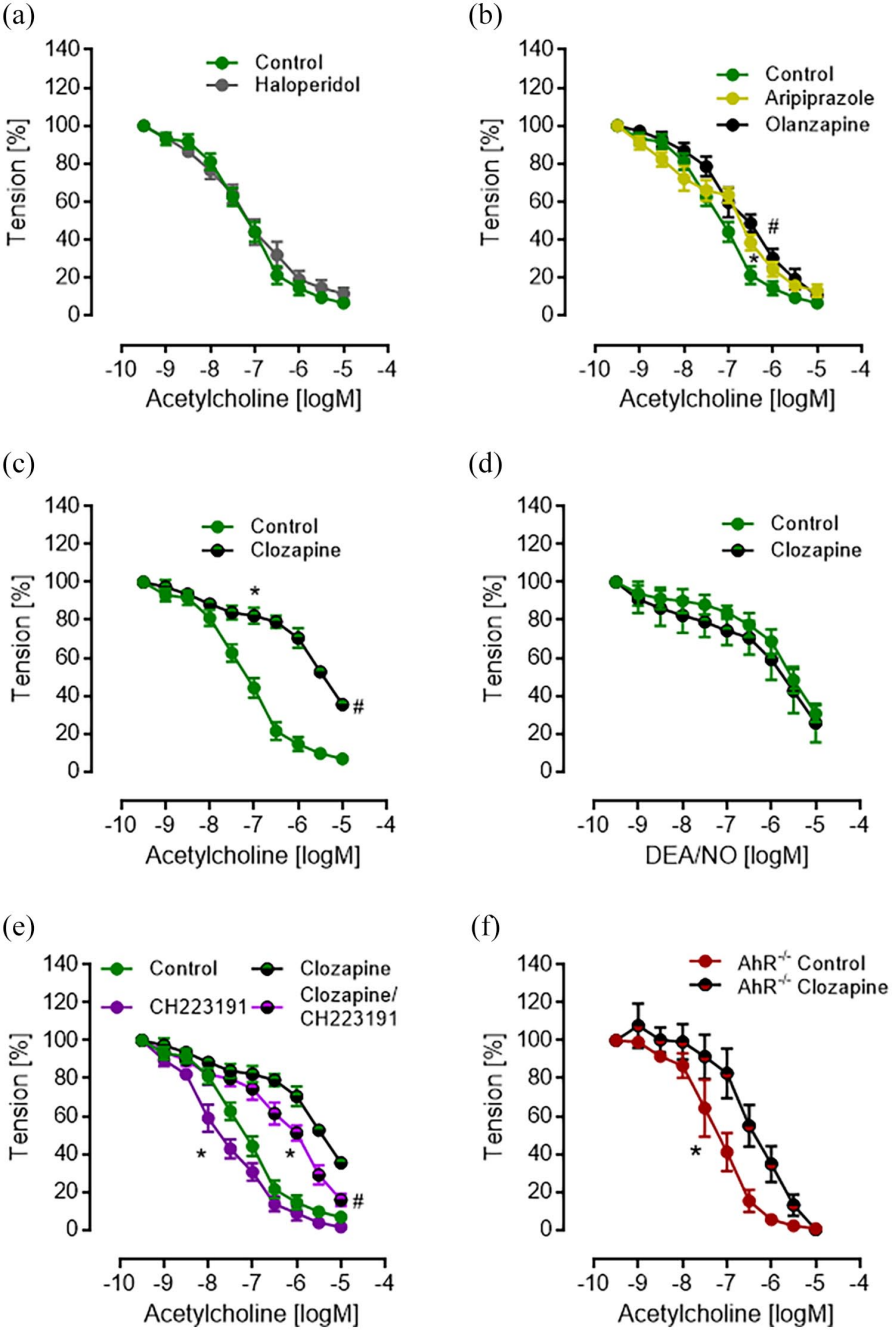
Concentration-dependent vasodilator response of thoracic aorta ring preparations in the absence (control) and presence of antipsychotic drugs (bars represent SEM). (a) Haloperidol (1 µM) showed no effect on endothelium-dependent vasodilation to acetylcholine (*n* = 6, *p* > 0.05, two-way ANOVA); (b) analysis by two-way ANOVA showed for 1 µM aripiprazole (*n* = 10, *p = 0.0199) and 0.5 µM olanzapine (*n* = 6, ^#^*p* = 0.0001) a small but significant rightward shift but no effect on maximal vasodilation (*p* > 0.05, Sidak multiple comparison test); (c) Clozapine (1 µM) induced a strong rightward shift of the concentration-response curve to acetylcholine (*n* = 8, *p < 0.0001, two-way ANOVA) and a significantly weaker maximal relaxation (^#^*p* < 0.0001, Sidak multiple comparison test); (d) Clozapine showed no effect on vasodilation to the NO-donor DEA/NO at any concentration (*n* = 6, *p* > 0.05, Sidak multiple comparison test following two-way ANOVA); (e) the aryl hydrocarbon receptor antagonist CH223191 (10 µM) significantly improved vasodilation (*n* = 6, *p < 0.0001, two-way ANOVA) and the maximal vasodilator response to acetylcholine in the presence of clozapine (^#^*p* = 0.0033, Sidak multiple comparison test). In addition, CH223191 improved vasodilation to acetylcholine in the absence of clozapine (*n* = 6, *p < 0.0001, two-way ANOVA), but did not alter the maximal vasodilator response (*p* > 0.05, Sidak multiple comparison test). (f) In contrast to C57BL6 mice, clozapine (1 µM) showed a lesser, albeit still significant (*p* < 0.001, two-way ANOVA), rightward shift of the concentration-response curve to acetylcholine in AhR-deficient mice (AhR^-/-^) and no effect on maximal vasodilation (*p* > 0.05, Sidak multiple comparison test).

Function of the endothelium was examined in aortic rings of male C57BL/6 mice by cumulative addition of acetylcholine after submaximal precontraction with prostaglandin F2α and 20 min incubation with APDs. While both dopamine receptor type 2 (D2R) antagonists haloperidol and aripiprazole did not influence vasodilatation (Figure 3(a) and (b)), olanzapine reduced vasorelaxation only slightly at 0.5 µM ACh (Figure 3(b)). In contrast, clozapine (1 µM) significantly reduced vasorelaxation with ACh from about 90% toward 65% (Figure 3(c)), although complete relaxation after clozapine incubation was achieved by cumulative concentrations of DEA/NO instead of Ach (Figure 3(d)). When AhR activation under clozapine was inhibited by CH22391, aortic rings were completely dilated by ACh despite clozapine preincubation, although a significant rightward shift of the concentration-response curve to ACh still remained (Figure 3(e)). AhR inhibition even improved vasodilation to acetylcholine in the absence of clozapine (Figure 3(e)). Aortic rings from AhR^-/-^ mice did not show reduced maximal vasorelaxation under clozapine, but again a significantly reduced sensitivity toward ACh (Figure 3(f)).

To further elucidate the effect size of clozapine on the vasodilator response to ACh we calculated the half-maximal vasodilator concentration (inhibitory concentration, IC50) of ACh given as pD2 values (-logM, higher values indicate higher efficacy). As compared with control conditions, neither aryl hydrocarbon receptor deficiency (AhR^-/-^) nor AhR inhibition by preincubation with CH223191 induced a change of ACh induced half-maximal endothelium-dependent vasodilation (Figure S2, n.s.). However, preincubation with clozapine (1 µM), clozapine plus CH223191 (10 µM), and clozapine in AhR-deficiency elicited a strong and significant reduction of ACh’s efficacy (one-way ANOVA, ^*^p < 0.001 vs control value, Tukey’s multiple comparison test). Likewise, clozapine slightly reduced the efficacy of ACh in AhR^-/-^-deficient mouse aortic rings as compared with transgene negative littermates (one-way ANOVA, ^#^*p* < 0.015 vs AhR^-/-^ control value, Tukey’s multiple comparison test). These data suggest a minor effect of baseline AhR activity on ACh induced endothelium-dependent vasorelaxation. In striking contrast, clozapine induced a strong inhibition of the vasodilator response to ACh which was for the most part dependent on activation of AhR.

## Discussion

The aim of this study was to investigate whether clozapine might interact with the cytosolic transcription factor AhR. Our new finding is that clozapine activates AhR signaling, while other APDs such as aripiprazole, haloperidol, and olanzapine show no AhR-stimulating activity. This effect of clozapine contributed to an increase of adipogenesis and elicited vascular endothelial dysfunction. These findings suggest that activation of AhR by clozapine might be associated with some of its well-known common side effects such as weight gain and hypertension (Alberich et al., 2019; Teff and Kim, 2011).

Clozapine-induced AhR activation occurred at the transcriptional as well as at the functional level. In order to show its direct, short-term effect, we used high clozapine concentrations in the luciferase reporter assays, but longer incubations under clinically more relevant concentrations of clozapine confirmed significant induction of the AhR target gene Cyp1A1 in human HepG2 cells and preadipocytes. The observed clozapine-mediated increase, although small when compared with activation by PAHs, was consistent and comparable to that of the chemotherapeutic drug doxorubicin (Volkova et al., 2011) and the p38 MAPK inhibitor SB203580 (Korashy et al., 2011). This activity of clozapine appears to be unlikely related to D2R antagonism which was previously shown to suppress AhR activation and Cyp1A1 expression (Harkitis et al., 2015). Accordingly, neither the full D2R antagonist haloperidol nor aripiprazole, an antagonist with partial agonistic activity, increased luciferase activity or reduced endothelium-dependent vasodilation in our experiments.

In a cell culture model, we previously differentiated human preadipocytes in the presence of APDs. Clozapine significantly increased adipogenesis (Hemmrich et al., 2006), while aripiprazole decreased adipogenic conversion (Hemmrich et al., 2011). Here we present evidence that this preadipocyte differentiation was triggered by AhR signaling, because clozapine-induced differentiation was strongly inhibited either by the AhR inhibitor α-NF or the green tea extract EGCG, which has AhR-antagonizing activities as well (Palermo et al., 2003, 2005). In line with our results, mice with an adipocyte-specific AhR gene deletion showed no weight gain under high fat diet (Gourronc et al., 2020). Increased glucose import together with decreased consumption by mitochondrial respiration leads to triglyceride accumulation in the preadipocytes (Vankoningsloo et al., 2005). The adipogenic cocktail applied to the preadipocytes simulates this condition. While dexamethasone inhibits mitochondrial respiration (Pandya et al., 2007), IBMX, insulin, thyronine, pioglitazone, and transferrin promote Akt (also known as protein kinase B) activation (Yun et al., 2019) and translocation of glucose transporters to the plasma membrane. The fact that pioglitazone promotes Akt activation even under insulin resistance (Yang et al., 2017) is important, because the preadipocytes were isolated from obese people with probable insulin resistance. Clozapine—like low levels of dioxin or PCB—increased preadipocyte differentiation and weight gain (Arsenescu et al., 2008; Hemmrich et al., 2006; Ortiz et al., 2013). Increased triglyceride accumulation in clozapine-treated 3T3L1 preadipocytes was independent from histamine H1 receptor and serotonergic 5’HT-2C receptor antagonism (Tsubai et al., 2017). Here we present evidence that instead AhR signaling is involved. The AhR target gene Cyp1B1 is located in the mitochondria and contributes to mitochondrial dysfunction (Bansal et al., 2014). This inhibition of mitochondrial respiration by clozapine (Scaini et al., 2018) together with increased production of reactive oxygen species (ROS) (Dobrocsyova et al., 2019) probably increased adipogenesis, which was suppressed by AhR inhibitors. Further studies will address this theme.

In addition to the AhR-dependent transcriptional effects induced by clozapine treatment, direct non-transcriptional AhR signaling by clozapine appeared to be evident by inhibition of endothelium-dependent vasodilation. Vascular endothelial cells are important regulators of a variety of vascular functions including vasomotor activity controlling organ blood flow, inhibition of platelet aggregation, monocyte adhesion and of apoptosis as well as antioxidant activities. The majority of these activities is supported by NO synthesized from L-arginine by the enzyme endothelial NO synthase (eNOS). The first observation evaluating the endocrine activity of vascular endothelial cells demonstrated that ACh induces an endothelium-dependent vasodilation (Furchgott and Zawadzki, 1980) and a similar effect was observed in humans (Ludmer et al., 1986). It became soon apparent that dysfunctional endothelium-dependent vasodilation occurs at early stages of many diseases such as hypertension, coronary artery disease, heart failure, atherosclerosis, and diabetes (Gewaltig and Kojda, 2002). However, the impairment of endothelial functions, which is generally referred to endothelial dysfunction, covers not only inhibition of endothelium-dependent vasodilation but typically defects in the generation and bioavailability of endothelial NO (Gimbrone and García-Cardeña, 2016).

Surprisingly, clozapine induced a strong inhibition of ACh-induced and NO-mediated endothelium-dependent relaxation in aortic rings from healthy mice, while aripiprazole and the structurally very similar olanzapine showed only small effects at concentrations related to the different serum concentrations of these drugs at therapeutically relevant doses. In addition, even a comparably high concentration of haloperidol showed no effect. The influence of clozapine was characterized by both a strong rightward shift of the concentration response curve and a remarkable suppression of the maximal vasodilator response resembling the typical reactivity of atherosclerotic arteries (Gewaltig and Kojda, 2002; Gimbrone and García-Cardeña, 2016). Further experiments using the AhR antagonist CH223191 suggested that activation of AhR by clozapine might contribute to endothelial dysfunction as it diminished the impairment of vasodilation to ACh at any concentration and largely improved the maximal vasodilator response. Likewise, in AhR-deficient mice no suppression of maximal vasodilation to ACh was observed. Of note, the effect of clozapine could not be attributed to an impairment of NO signaling because it did not alter vasodilation to the NO-donor DEA/NO. These data suggest an inhibitory effect of clozapine on the activity of eNOS in mouse aortic endothelium which involves activation of the AhR.

It is well known that ACh-induced endothelium-dependent aortic vasorelaxation is exclusively mediated by activation of eNOS and that an increased activity of AKT is crucially involved (Luo et al., 2000). It has been shown as well that AhR activation by 3-methylcholanthrene which increased blood pressure in mice reduced eNOS phosphorylation through the AhR/RhoA-mediated inactivation of Akt1 (Chang et al., 2017). In line with these results, endothelial Akt1 ablation in transgenic mice promoted hypertension and endothelial dysfunction (Lee et al., 2018). In addition, the results of previous reports suggest that clozapine also inhibits AKT (Chen et al., 2011; Han et al., 2017; Shin et al., 2006), but so far a direct link to activation of the AhR was not shown. Therefore, reduced endothelial NO production under clozapine—reported recently by Nair et al. (2019)—might be triggered by AhR activation leading to decreased AKT activity and inhibition of eNOS.

However, there was still an impairment of ACh-induced vasodilation in aortic rings of AhR^-/-^ mice. It is very well known that clozapine has strong antagonistic effect at several receptors including muscarinic acetylcholine receptors (Olianas et al., 1999) and these G-protein coupled receptors, particularly the subtypes 1 and 3, are expressed in endothelial cells and translate the signal of ACh to induce activation of NO production via Gq coupling (Bény et al., 2008; Khurana et al., 2004). Hence, it appears reasonable to assume that the anti-muscarinic activity of clozapine contributed to the impairment of ACh-induced vasodilation in AhR-deficient and C57BL6 control mice. Regarding the magnitude of the rightward shift of the ACh concentration response curve in aortic rings of AhR^-/-^ mice, other potentially operating mechanisms such as increased oxidative stress (Nair et al., 2019) and/or endoplasmatic reticulum stress (Lauressergues et al., 2012; Murugan et al., 2015) are likely involved. Therefore, further investigations to elucidate the influence of clozapine on endothelial NO-generation in response to ACh are necessary to elucidate this potentially novel activity.

Activation of AhR has been associated with increased incidence of hypertension. For example, coke oven workers are exposed to high concentrations of PAH which leads to hypertension and abnormal electrocardiography (Liang et al., 2016). In young cigarette smokers impaired flow-mediated dilation was associated with increased serum PAH levels resulting in increased AhR activity (Wiest et al., 2015). However, activation of the AhR with subsequent induction of antioxidative defense mechanisms reduced heart failure in hypertensive rats (Seymour et al., 2013).

In patients diagnosed with schizophrenia, short-term treatment (3 days) with clozapine induced transient hypotension (Parks et al., 2018), most likely due to direct α1 adrenergic receptor antagonism. During 8 weeks of clozapine treatment, heart rate increased and blood pressure shifted toward hypertension (Norman et al., 2017). Although there is no evidence from clinical studies, one might speculate that hypertension in response to long-term clozapine treatment might be linked to AhR signaling. Here we present evidence that clozapine activates the AhR pathway. Reduced vasodilatation and increased adipogenesis under clozapine point to physiological effects of AhR activation in mice and humans.

Our findings may help to elucidate some of the actions of clozapine that have not been commonly recognized so far. On one side, a possible concern for psychiatrists and their patients is whether AhR activation is involved in the side effects of clozapine treatment like agranulocytosis, hypertension, or weight gain. Since exposure to environmental PAHs can cause insulin resistance, abdominal obesity (Lee et al., 2011) and leukopenia (Lan et al., 2004), too, one might attribute the adverse effects of clozapine—probably in synergy with other AhR ligands—to AhR activation. On the other side, AhR activation is involved in a number of detoxifying and cytoprotective pathways that enhance antioxidative defense mechanisms (Wang et al., 2018). Since oxidative stress is higher in patients diagnosed with schizophrenia (Wu et al., 2013), beneficial effects of clozapine treatment might also result from AhR signaling.

Finally, further studies should investigate clozapine’s effects beyond the vasculature and adipogenesis, for example, its effects on the brain via AhR signaling. The AhR is expressed in neuronal progenitor cells in the hippocampus (Latchney et al., 2013). In AhR^-/-^ mice, brain aging and loss of the white matter integrity was more pronounced than in age-matched wild-type mice (Bravo-Ferrer et al., 2019), thereby implicating protective effects of AhR signaling in the brain. Likewise, neuroprotective activities of clozapine as shown in the Tg-APPswe/PS1dE9 mouse model of Alzheimer’s disease (Choi et al., 2017) might also be related to increased AhR signaling.
